# Tumor budding - a potential biomarker in low grade salivary gland carcinomas?

**DOI:** 10.3389/fonc.2024.1410264

**Published:** 2024-06-25

**Authors:** Valentin Burkhardt, Gian Kayser, Theo Villing, Christoph Becker

**Affiliations:** ^1^ Department of Oto-Rhino-Laryngology, Medical Center – University of Freiburg, Faculty of Medicine, University of Freiburg, Freiburg, Germany; ^2^ Institute of Pathology Naehrig Mattern Kayser, Freiburg, Germany

**Keywords:** tumor budding, low grade salivary gland carcinoma, salivary gland carcinoma, cancer, prognostic factor, biomarker

## Abstract

**Background:**

Low-grade salivary gland carcinoma is regularly treated with surgical therapy of the salivary gland without elective neck dissection in T1/2 carcinomas, either alone or with adjuvant radiation therapy. However, occult metastasis and locoregional recurrence influence therapy and outcome. Tumor budding is an emerging prognostic pathological factor in many carcinomas, but has not yet been adequately considered in salivary gland carcinomas.

**Methods:**

We conducted a retrospective single-center study of 64 patients diagnosed with low-grade carcinoma of the major salivary glands treated between 2003 and 2017. Pathological risk factors and TNM classification were thoroughly assessed for each case. All hematoxylin and eosin (HE)-stained histological specimens underwent careful examination, and tumor budding was identified following the guidelines set forth by the International Tumor Budding Consensus Conference in 2016.

**Results:**

Tumor budding was not statistically significant concerning 5-year survival rate (5-YSR) (*p*=0.969) and mean overall survival (log-rank *p*=0.315). Whereas 5-year disease-free survival rate (5-YDFSR) was 87% in the low tumor budding group and 61.1% in the intermediate and high tumor budding group (*p*=0.021). Mean disease-free survival accounted for 100.2 months (CI: 88.6;111.9) in the low budding score group and 58.7 months (CI: 42.8;74.6) in the other group (log-rank *p*=0.032). Notably, pT1/2 showed significantly lower tumor buds than pT3/4 stages (2.43 tumor buds/0.785 mm^2^ vs. 4.19 tumor buds/0.785 mm^2^, *p*=0.034). Similar findings were noted comparing nodal-positive and nodal-negative patients, as well as patients with and without lymphovascular invasion and perineural invasion (each *p*<0.05).

**Conclusions:**

Tumor budding might be used as an additional prognostic factor for recurrence in low-grade salivary gland carcinoma, seemingly associated with a higher nodal metastasis rate and advanced tumor stages and a worse 5-YDFSR. Consequently, the evaluation of tumor budding in resection specimens of low-grade salivary gland tumor may prove valuable in decision-making for neck dissection and follow-up strategy.

## 1 Introduction

Salivary gland tumors manifest themselves in various entities, localizations and morphologies. They contribute 3 – 10% of all head and neck tumors, making them relatively rare. The estimated incidence ranges from 0.4 to 13.5 cases per 100,000 annually in the United Kingdom (1). Given their low incidence, comprehensive studies with substantial numbers of cases are rare. The World Health Organization (WHO) lists 39 salivary gland pathologies in the current 5th WHO Classification of Tumors. These are categorized into four groups: mesenchymal tumors specific to the salivary glands, malignant epithelial tumors, benign epithelial tumors and non-neoplastic epithelial lesions (2). While most tumors in major and minor salivary glands are benign, up to 35% of the lesions prove malignant, exhibiting varied distributions among the different salivary glands (1, 3). The most prevalent malignant entities are the mucoepidermoid carcinoma (MEC) and the adenoid cystic carcinoma (ACC) (4).

Grading plays a pivotal role in determining subsequent therapeutic interventions in the contemporary management of major salivary gland carcinoma (MSGC) (5, 6). Due to the diverse histogenetic, biological, prognostical and phenotypical properties of these carcinomas, a uniform grading system is lacking. Consequently, two grading strategies have emerged. One group of malignant tumors is classified due to its entity-related characteristics as low- or high-grade, respectively. For instance, acinic cell carcinomas and basal cell adenocarcinomas are consistently classified as low-grade, while lymphoepithelial carcinomas and salivary duct carcinomas fall into the high-grade category by definition. The other strategy involves grading entities like mucoepidermoid carcinoma, adenocarcinoma and adenoid cystic carcinoma based on their cytological and histological appearance (2, 7–11).

Tumor budding has gained interest across various medical disciplines in the current discourse on prognostic biomarkers of tumor entities. First described by Imai et al. in the 1950s (12), tumor budding is now well-established in colorectal cancer as an independent prognostic biomarker. Tumor buds are defined as single-cell nests of cancer cells at the invasive tumor front, with up to four cells in total (13). Tumor buds are part of the tumor microenvironment and are associated with processes of epithelial-mesenchymal transition (EMT) (14, 15). The tumor budding described at the invasive front must be distinguished from intratumoral budding (16). While there are indications of a correlation between increased tumor buds and tumor progression in head and neck squamous cell carcinoma (HNSCC) (17), the impact of tumor buds in MSGC has not been systemically explored. This study aims to analyze the influence of tumor budding in low-grade MSGC on metastasis, tumor size, recurrences, overall survival and disease-free survival. Insights into survival and recurrences patterns are clinically significant and could potentially alter therapy regimens for patients.

## 2 Material and methods

### 2.1 Retrospective analysis

For the retrospective analysis, data were gathered from all MSGC patients treated in the ENT department of the University Medical Center of Freiburg. The inclusion criteria encompassed all patients newly diagnosed with low-grade MSGC between 2003 and 2017 at the ENT department of the University Medical Center of Freiburg. Survival and recurrence data were sourced from the National Tumor Register and the Comprehensive Cancer Center Freiburg, totaling 78 patients. 14 patients had to be excluded due to insufficient specimen material, extreme inflammation on tumor-host interface and missing availability of specimen in our electronic pathological register Patho-Pro (RC-Modus, Mai 2019, Version: 9.0.9070, OS-Version 6.1, Java-Version 1.7). Tumor budding was established in 64 patients. All patients underwent curative-intent surgery. The histological glass slides from these surgical specimens, archived at the Institute of Surgical Pathology, University Medical Center Freiburg, were retrieved and reviewed to verify diagnoses and tumor stages.

This study was approved by the Ethics Committee of the Albert-Ludwigs-University Freiburg (Ethics Commission number 176/18) and registered at the German Clinical Trials Register number is DRKS00015825.

### 2.2 Tumor bud survey

For each patient, two hematoxylin-eosin (H&E) stained tissue slides were digitized (Panoramic Scan, 3D Histech, Budapest, Hungary) and afterwards processed using the QuPath software (Version 0.1.2). QuPath is an open source software, facilitating analyses of digital microscopic sections (18). Functions like area measurement and cell marking helps assessing tumor buds and avoiding mistakes such as double counts. Tumor budding was afterwards counted on both slides as recommended by the International Tumor Budding Consensus Conference (ITBCC) (19). Tumor buds were defined as tumor cell nests with up to 4 cells. In each tumor bud, only tumor cells are noted and must be distinguished from superimposed cell nuclei of lymphocytes or stromal proliferations. However, tumor cells displayed more prominent nucleoli and were distinguished from stromal proliferations by stronger cytoplasmic staining.

**Figure 1:**
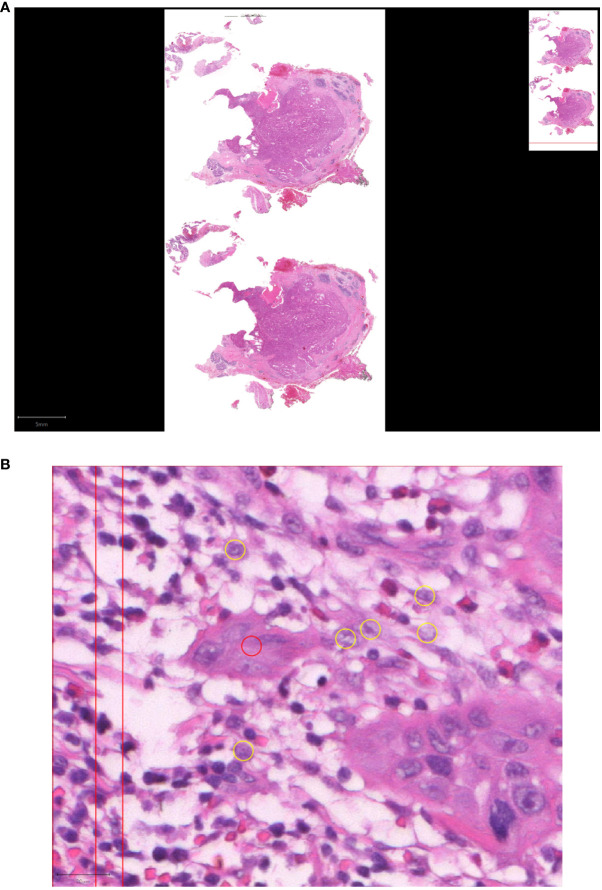
Tumor budding in low-grade MSGC. **(A)** Digitalized specimen of MSGC. **(B)** Tumor budding in HE stained SGC slides was analyzed using QuPath at 20-fold magnification at the tumor host interface to reveal the morphology of the budding cells as a cell cluster (≤ four tumor cells, red circle) separated from the main tumor mass (yellow circles).

Subsequently, tumor bud hotspots were evaluated in 10-fold magnification, the area with the highest tumor bud amount (hotspot) was used for analyses. Subsequently, tumor buds at the peritumoral invasion front were counted in 20-fold magnification (**Figure 1**). Using QuPath, 10 randomly inserted rectangles covering areas of 0.785 mm^2^ were placed over the hotspot of the tumor invasion front. To generate the tumor buds per 0.785 mm^2^, a mean value of the two included specimens of each patient was calculated. Tumor budding was categorized into two groups: low tumor budding with ≤4 tumor buds and high tumor budding with >4 tumor buds per 0.785 mm^2^. This adjustment was made following the recommendations of the ITBCC (19). Due to the limited number of patients, we did not differentiate between intermediate and high tumor budding.

The tumor-host interface was determined only in the periphery of the main tumor tissue surrounded by other tissue, regardless of whether it is salivary gland tissue, fat, fibrosis or connective tissue. If placed at the edge of the specimen, slides were excluded, because there could have been cutting off the tumor. Assessment was difficult in areas of extreme inflammation and, in some cases, led to exclusion of the specimen.

### 2.3 Statistical analysis

Given their reference to different subsets, the primary questions were not adjusted for multiple testing. Prior to testing, all variables underwent evaluation for normal distribution with the Shapiro-Wilk test. Statistical methods for analysis included t-test and Mann-Whitney-U tests for mean comparison. Overall survival (OS) was defined as the period between primary surgery and last contact to the patient, while disease-free survival (DFS) was defined as the period between primary surgery and the detection of a recurrence. Survival analysis included Kaplan-Meier curves, Log-rank tests as well as Cox proportional hazards regression and likelihood ratio χ2-tests (LRχ2). The Kendall-Rank-Correlation was used to calculate the correlation between compiled tumor budding and clinicopathological factors. Hazard ratios (HR) with associated 95% confidence intervals (CI) and *p*-values were used to estimate the risk. A *p*-value <0.05 was defined as statistically significant for all analyses. Data analysis was conducted using IBM SPSS Statistics 29; (IBM, Armonk, New York, USA).

## 3 Results

### 3.1 Patient cohort

A total of 64 patients were included, comprising 31 male and 33 female patients. The mean age at first diagnosis was 59.8 years, with a median follow-up duration of 96.8 months (CI: 80.4;113.1). Recurrence was observed in 15 patients. All patients underwent primary surgery. 54 (84.4%) tumors were located in the parotid gland, 9 (14.1%) in the submandibular gland and 1 (1.6%) in the sublingual gland.

Estimated mean OS in Kaplan-Meier analysis was 117.3 months (CI: 98.4;136.2) and mean DFS was 92.9 months (CI: 81.6;104.2).

**Figure 2:**
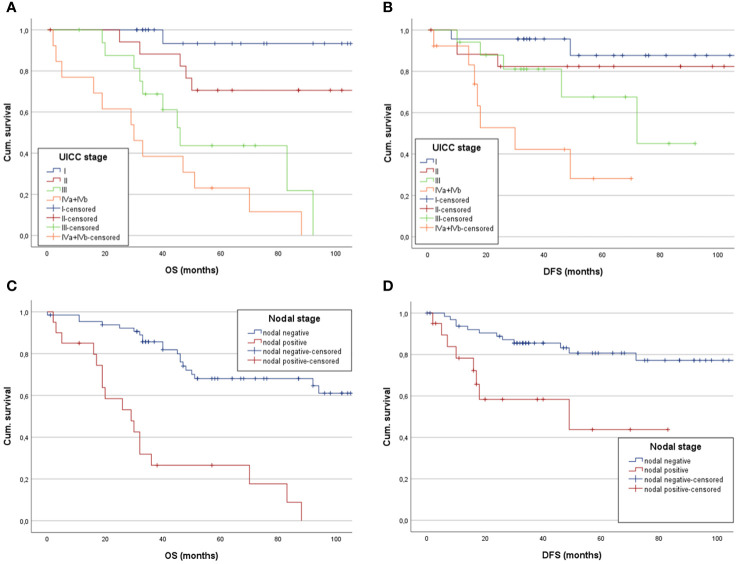
Estimated mean OS and DFS of low-grade SGC patients analyzing the impact of **(A)** UICC stages, showing a statistically significant decreased mean OS in patients with higher UICC stages, **(B)** as does the Kapan-Meier analysis for mean DFS separated between the UICC stages. **(C)** Kaplan-Meier showing a significantly longer mean OS for patients without nodal positive neck and **(D)** demonstrating a significantly longer mean DFS for patients with nodal negative neck.

Kaplan-Meier analysis of OS stratified by UICC stage demonstrated 163.2 months (CI: 139.6;186.8) in UICC I, 95.8 months (CI: 78.6;113.1) in UICC II, 63.7 months (CI: 45.6;81.8) in UICC III and 39.9 months (CI: 18.8;61) in UICC IV, respectively (**Figure 2A**). Log-rank testing revealed a statistically significant difference for UICC stage in mean OS (each *p*<0.001). Kaplan-Meier analysis of mean DFS by UICC stages showed similar results (UICC I: 100.7 months, CI: 88.5;112.9. UICC II: 98 months, CI: 79;116.9. UICC III: 69.7 months, CI: 52.1;87.3. UICC IV: 33.5 months, CI: 19.5;47.5. log-rank test: *p*=0.002, **Figure 2B**). Comparing nodal positive and nodal negative patients, 5-YSR was 45,5% in nodal positive patients and 77.8% in nodal negative patients (*p<*0.029). Mean OS with Kaplan-Meier analysis showed 45.5 months (CI: 22.9;68.2) and 132.4 months (CI: 113.4;151.3), respectively (**Figure 2C**). Log-rank testing resulted in a *p*-value of <0.001, indicating statistically significant differences for nodal status at primary diagnosis and mean OS. 5-YDFS was 81.5% in nodal negative patients and 72.7% in nodal positive patients (*p=*0.03). Estimated mean DFS in nodal positive patients was 51.9 months (CI: 29.5;74.2) and 97.2 months (CI: 86;108.5) in nodal negative patients (log-rank: *p*=0.042) (**Figure 2D**).

### 3.2 Survival analysis for tumor budding

The population was divided into two groups: a low budding score (≤4 tumor buds/0.785 mm^2^) and high budding score (> 4 tumor buds/0.785 mm^2^). Of the total, 46 patients (71.9%) had a low budding score and 18 patients (28.1%) had a high budding score (**Table 1**). In the low budding score group 9 patients (19.6%) experienced recurrence compared to 6 patients (33.3%) in the high budding score group. The low tumor budding group contained more UICC I/II patients than the high tumor budding group (**Table 1**). During follow-up, 14 patients (30.4%) in the low tumor budding and 8 patients (44.4%) in the high tumor budding group died (**Table 1**). There were no statistically significant differences between the two groups regarding gender, age at first diagnosis, tumor entities and UICC stage.

**Table 1:** The table offers an overview of the numbers of patients in the low and high tumor budding group for age at diagnosis, sex, location of the carcinoma, entity, pT/N stage, UICC stage, recurrences and death.

	Low tumor budding	High tumor budding	*p* value
N	46	18	
Age at initial diagnosis (years)			0.35
Mean ± SD (Median)	60.6 ± 2.69 (63)	59 ± 4.99 (63)	
Gender, n (%)			0.339
male	24 (52.2)	7 (38.9)	
female	22 (47.8)	11 (61.1)	
Location, n (%)			0.124
Parotid gland	41 (89.1)	13 (72.2)	
Submandibular gland	4 (8.7)	5 (27.8)	
Sublingual gland	1 (2.2)	0 (0)	
Entity, n (%)			0.358
Mucoepidermoid carcinoma	11 (23.9)	2 (11)	
Adenocarcinoma, NOS	8 (17.4)	6 (33.3)	
Adenoid cystic carcinoma	6 (13)	4 (22.2)	
Acinic cell carcinoma	10 (21.7)	3 (16.7)	
Intraductal carcinoma	1 (2.2)	2 (11.1)	
Myoepithelial carcinoma	2 (4.3)	0 (0)	
Carcinoma ex PA	0 (0)	1 (5.6)	
Others	8 (17.4)	0 (0)	
pT Stage, n (%)			0.242
T 1	19 (41.3)	5 (27.8)	
T 2	16 (34.8)	4 (22.2)	
T 3	8 (17.4)	7 (38.9)	
T 4	3 (6.5)	2 (11.1)	
pN Stage, n (%)			0.174
N 0	33 (71.7)	9 (50)	
N 1	2 (4.3)	3 (16.7)	
N 2	1 (2.2)	0 (0)	
N 3	2 (4.3)	3 (16.7)	
No Neck dissection performed	8 (17.4)	3 (16.7)	
M Stage, n (%)			
M 0	46 (100)	18 (100)	
M 1	0 (0)	0 (0)	
UICC Stage, n (%)			0.062
UICC I	19 (41.3)	4 (22.2)	
UICC II	15 (32.6)	3 (16.7)	
UICC III	8 (17.4)	6 (33.3)	
UICC IVA/B	4 (8.7)	5 (27.8)	
Recurrence, n (%)			0.242
No recurrence	37 (80.4)	12 (66.7)	
Recurrence	9 (19.6)	6 (33.3)	
Death, n (%)			0.289
Alive	32 (69.6)	10 (55.6)	
Dead	14 (30.4)	8 (44.4)	

**Figure 3:**
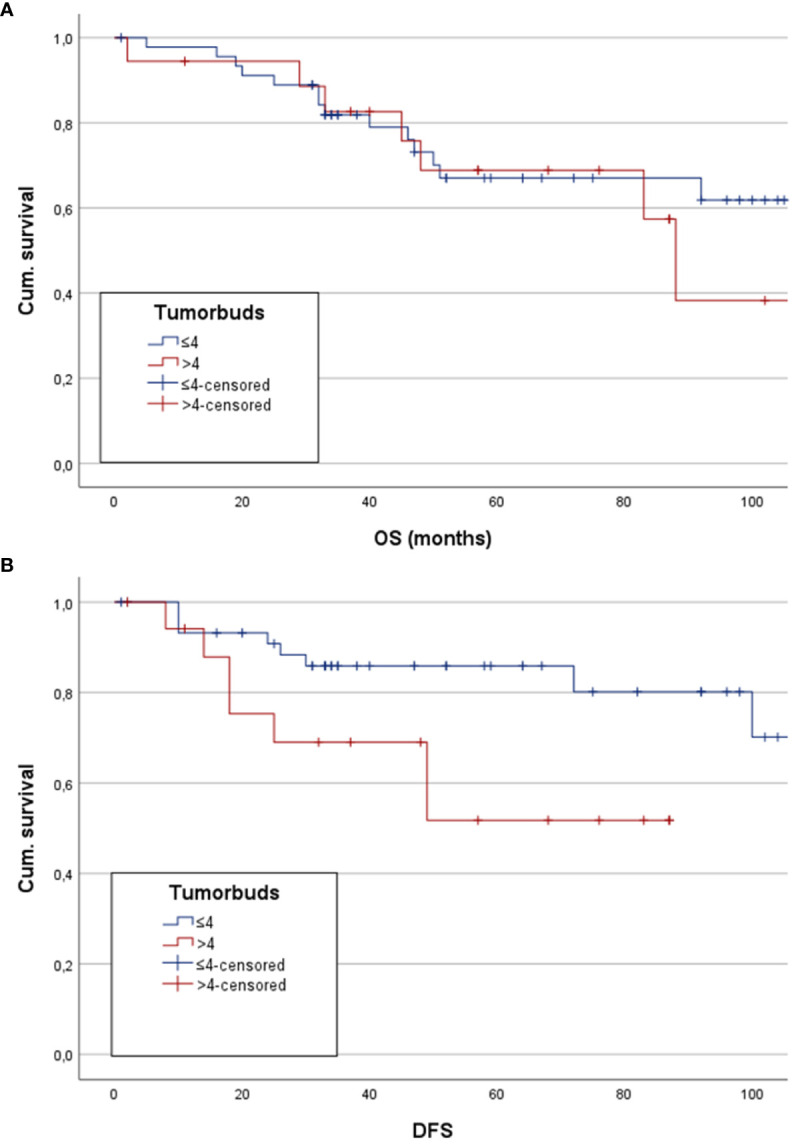
Here estimated mean OS and DFS for patients with low and high tumor budding are depicted as Kaplan-Meier analysis and Hazard ratio, respectively. **(A)** Kapan-Meier analysis shows no statistically significant difference between the groups. **(B)** Depiction of a significantly higher mean DFS in patients with low tumor budding score.

5-YSR was 71.7% in the low tumor budding group and 72.2% in the high tumor budding group, which accounts for no significant difference between the two groups (χ2 *p=*0.969). The comparison of mean OS showed 89.5 months (CI: 76.8;102.2) in the low budding score group and 102.3 months (CI: 62.9;141.8) in the high budding score group (*p*=0.575) (**Figure 3A**). Cox regression analysis showed no significant correlation between a higher budding score and mean OS (*p*=0.576, hazard ratio (HR): 1.297; 95% confidence interval (CI): 0.52;3.23).

5-YDFSR was 87% in the low tumor budding group, whereas the high tumor budding group reached a 5-YDFSR of 61.1%. There was a statistically significant difference between the two groups (χ2 *p=*0.021). Kaplan-Meier analysis of estimated mean DFS showed 100.2 months (CI: 88.6;111.9) in the low budding score group and 76.5 months 58.7 months (CI: 42.8;74.6) in the other group. The log-rank test revealed a statistically significant difference between the groups (*p*=0.032) with longer mean DFS in the low budding score group (**Figure 3B**). On the other hand, Cox regression analysis showed a statistically significant worse mean DFS in the high tumor budding group (*p*=0.043, HR: 2.954, CI: 1.035;8.434).

### 3.3 Tumor budding analysis

**Figure 4:**
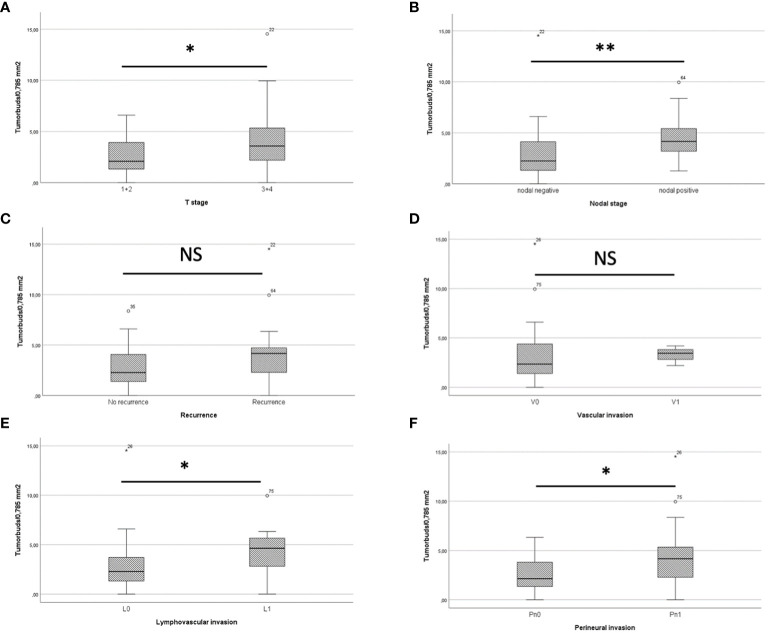
**(A)** shows a boxplot comparing tumor buds in pT1/2 and pT3/4 patients, showing a significant difference. In boxplot **(B)** Tumor buds in patients with and without recurrence are compared, also showing a statistically significant difference. **(C)** The boxplot depicts that patients with recurrence do have more tumor buds than patients without recurrence. **(D)** There was no significant difference in mean tumor buds/0.785mm^2^ between the group of patients with and without vascular invasion. The Boxplots **(E)** for lymphovascular invasion and **(F)** for perineural invasion both show a significantly higher tumor budding in patients with lymphovascular/perineural invasion. * = *p*<0.05. ** = *p*<0.01. NS = not significant.

In comparison of pT1/2 and pT3/4 stages, there was a mean of 2.43 ± 1.46 tumor buds/0.785 mm^2^ in pT1/2 carcinomas and 4.19 ± 3.5 tumor buds/0.785 mm^2^ in pT3/4 carcinomas, resulting in a statistically significant difference in Mann-Whitney-U test analysis (*p*=0.034) (**Figure 4A**). Nodal negative patients had a mean of 2.62 ± 2.25 tumor buds/0.785 mm^2^, whereas nodal positive patients had a mean of 4.72 ± 2.52 tumor buds/0.785 mm^2^ (**Figure 4B**). Mann-Whitney-U test analysis yielded a statistically significant difference between the two groups (*p*=0.004). The comparison of patients with and without recurrence showed 4.15 ± 3.67 tumor buds/0.785 mm^2^ and 2.62 ± 1.78 tumor buds/0.785 mm^2^, respectively (**Figure 4C**). Mann-Whitney-U test analysis revealed no statistically significant difference (*p*=0.14). Patients with lymphangioinvasion showed a mean of 4.27 ± 2.7 tumor buds/0.785 mm^2^, whereas a mean of 2.6 ± 2.23 tumor buds/0.785 mm^2^ was detected in patients without lymphangioinvasion (*p*=0.022, **Figure 4D**). There was no statistically significant difference in mean tumor buds between the groups of patients with vascular invasion vs. without vascular invasion (*p*=0.433, **Figure 4E**). The mean tumor budding differed significantly between patients, who had perineural invasion (mean of 4.5 ± 3.76 tumor buds/0.785 mm^2^) vs. those without perineural invasion (mean of 2.47 ± 1.52 tumor buds/0.785 mm^2^, *p*=0.03, **Figure 4F**).

### 3.4 Kendall-Rank-Correlation

The Shapiro-Wilk-Test revealed no normal distribution for the comprised tumor budding. Therefore, Kendall-Rank-Correlation was carried out to analyze correlation between tumor budding and clinicopathological risk factors.

Statistically significant positive correlation between tumor budding and N-Stage (*p*=0.019), UICC stage (*p*=0.021), lymphangioinvasion (*p*=0.022) and perineural invasion (*p*=0.03) were found (**Table 2**).

**Table 2:** Kendall-Rank-Correlation of tumor budding and clinicopathological factors, showing the parameter, Kendall-Tau and *p*-value.

Parameter	Kendall τ	*p-*value
**Age at initial diagnosis**	-0.046	0.594
**Sex**	-0.047	0.653
**Location**	0.140	0.175
**T-Stage**	0.174	0.071
**N-Stage**	0.237	0.019
**UICC-Stage**	0.221	0.021
**Lymphangioinvasion**	0.244	0.022
**Vascular Invasion**	0.087	0.414
**Perineural Invasion**	0.227	0.03
**Recurrence**	0.153	0.14

## 4 Discussion

This retrospective exploratory study is the first to explicitly evaluate the influence of tumor budding on 5-YSR, 5-YDFSR, mean OS and DFS in MSGC. As an emerging prognostic factor in tumor diagnosis and therapy, tumor budding is gaining attention in several solid tumors. The prognostic function of tumor budding in HNSCC (head and neck squamous cell cancer) has been discussed since the 2010s (20). However, there has been no published study analyzing tumor budding as a prognostic factor for MSGC until now.

The ongoing debate on less extended surgical procedures for MSGCs presents both benefits and risks for patients (21, 22). Undoubtedly aggressive surgical procedures such as total or radical parotidectomy, neck dissections and resection of the facial nerve, may result in impairment of quality of life and a higher morbidity (21). In this study 15 (23.4%) of low-grade MSGC patients experienced recurrence, emphasizing the need for identifying risk factors to tailor specific therapy regimens. These findings suggest that there is an unmet need to improve locoregional control of MSGC. Tumor budding might serve as the prognostic factor for locoregional recurrences to improve the therapy of MSGC.

Gaining a better understanding of the locoregional recurrence of low-grade MSGC presupposes a better understanding of the multistep process of carcinogenesis. It involves invasion and metastasis, clinically important hallmarks in cancer prognosis and treatment. Tumor budding, characterized by the loss of cell-cell contact and increased mobility, is thought to play a pivotal role in these processes across various cancer types (13, 14, 23). Higher tumor budding is correlated with advanced T- and N-classifications and poorer OS and DFS in colorectal carcinoma (19), ductal carcinoma of the breast (24), cholangiocellular carcinoma (25), nasopharyngeal carcinoma (26) and oral squamous cell carcinoma (27). While tumor budding serves as an additional prognostic marker in colorectal carcinoma, its implementation in head and neck cancer, including MSGC, has not yet become established.

The underlying mechanisms of tumor budding remain incompletely understood, with recent suspicions pointing toward epithelial-mesenchymal transition (EMT) as the potentially underlying mechanism. EMT, a developmental program, is associated with increased motility, resistance to apoptosis and higher invasiveness (13). Tumor budding seems to be a dynamic process in which the ‘hybrid’ EMT phenotype of buds with downregulation of epithelial (e.g. cytokeratin) and upregulation of mesenchymal (e.g. vimentin) molecules causes further invasion into tissue (28). The mechanism of EMT is the subject of current research in salivary gland carcinomas. There is evidence that EMT is associated with higher invasiveness and therefore a worse prognosis (29, 30). Nevertheless, there is no evidence for a link between EMT and tumor budding in salivary gland cancer yet. The assumption of a more aggressive cancer and therefore higher metastasis and more frequent recurrences due to high tumor budding is currently under debate in many solid cancers.

In this study, the evaluation and reporting of tumor budding was adapted to the current recommendations of the ITBCC (19). Due to the small number of patients included in our study, the existing tumor budding score was modified into a two-group score: low tumor budding (0–4 buds/0.785 mm^2^) and high tumor budding (>4 buds/0.785 mm^2^). The analysis focused on peritumoral tumor buds (PTB) at the invasion front, aligning with classical reporting for tumor buds as recommended in colorectal carcinoma (19). Nevertheless, evidence suggests that intratumoral tumor buds (ITB) may correlate with higher T- and N-stages in colorectal carcinoma and intrahepatic cholangiocellular carcinoma, making them useful in biopsy specimens (16, 25).

The results of our study indicate that there might be a need for further locoregional control in the subgroup of low-grade MSGC with a high tumor budding score, as patients in this group showed a lower 5-YDFS (61.1% vs. 87%, *p=*0.021) and mean DFS. However, the current American Society of Clinical Oncology (ASCO) guideline recommends a limited local surgical approach for T1/2 low-grade parotid gland cancers, reserving total or subtotal parotidectomy for high-grade cancers or advanced local spread (T3/4) (31). Controversies also exist regarding neck dissection in cN0 patients (21, 22). These guidelines show a trend toward surgical de-escalation of MSGC treatment. In order to avoid undertreatment, identifying potential risk factors beyond the established ones becomes crucial for further individualization of oncological management.

The parotid gland emerged as the most common location for MSGC in the current study, consistent with prior reports (25). Furthermore, our analysis demonstrated a significant decrease in OS for higher UICC stages, aligning with findings of other research groups (32–35). Nodal metastasis significantly lowers 5-year survival, as reported by Meyer and colleagues (35). This correlates with our data, showing mean OS of 45.5 and 132.4 months in nodal positive vs. nodal negative patients. At the current state, poor outcome of patients with MSGC is strongly associated with tumor stage, grading, lymph node metastasis and age (36). However, there is still a need for identification of further risk factors to achieve a better prognosis and differentiation in such patients.

Tumor grading has already been established as a prognostic factor and therefore directly affects the therapy regimen of MSGC. High-grade MSGC typically entails surgical resection, elective neck dissection and adjuvant radiation therapy (RT). Conversely, low-grade MSGC without nodal metastasis often achieves excellent OS and DFS with resection alone (31, 33). Elective neck dissection seems to be favorable for patients with T3/4 tumors and all high-grade MSGC (31, 34). However, our data indicate that there are also low-grade MSGCs with high tumor budding that lack locoregional control, as evidenced by the lower 5-YDFSR. The publication by Armstrong et al. demonstrated an increased occult lymph node metastasis in high-grade carcinoma, leading to the need for elective neck dissection to improve OS and DFS (37). The rate of occult metastasis differs in the literature. The Ketterer at al. study from 2019 did not find any occult metastases in cN0 staged MSGC (38). Concordant with decision-making for elective neck dissection, adjuvant RT of the ipsilateral neck is not routinely performed in low-grade MSGC (39–41). The data of both Ali et al. and Park et al. indicate a need of further local control in low-grade MSGC patients with locally advanced carcinomas, pathological risk factors and nodal metastasis (34, 42).

The findings in the current study show a significantly higher rate of recurrence and more lymph node metastasis in low-grade MSGC patients with high tumor budding. Therefore high tumor budding might add another level besides grading, tumor size and locoregional metastasis to take into consideration while planning the therapy of MSGC. Recurrences occurred in 19.6% of the low tumor budding group compared to 33.3% of the high tumor budding group. Furthermore, Kaplan-Meier analysis of mean DFS revealed 100.2 months in the low tumor budding score group and 58.7 months in the high tumor budding group (log-rank test *p*=0.032). Tumor budding correlated with UICC-stage (*p=*0.021), N-stage (*p=*0.019), lymphangioinvasion (*p=*0.022) and perineural invasion (*p=*0.03). These observations suggest a potentially more aggressive tumor infiltration, adding another feature to the considerations for patients with MSGC.

In 2019 Nakaguro et al. proposed a novel histologic risk stratification for salivary duct carcinoma. The risk stratification includes pathological factors such as prominent nuclear pleomorphism, mitosis ≥30/10 HPF, vascular invasion, high tumor budding and poorly differentiated clusters. The retrospective analysis demonstrated a significant impact of tumor budding on OS. DFS was not examined in their study (43). The current study, however, did not find a significant difference in 5-YSR or mean OS for patients with high tumor budding scores. The difference in findings may be attributed to variations in the number of patients (151 vs. 64) and the specific entity of salivary gland carcinoma included in the study by Nakaguro. Furthermore Nakaguro et al. used a different cut-off with 10 tumor buds/0.785 mm^2^, not the 4 tumor buds/0.785 mm^2^ that were used in our study. However, our findings indicate that tumor budding may be a factor of high interest in the treatment strategy for low-grade MSGC. It is associated with a worse 5-YDFSR, shorter mean DFS and correlates with higher N-stages, UICC-stages, lymphangioinvasion and perineural invasion. It might serve as a pathological risk factor, influencing decisions about neck dissection and adjuvant RT.

The presented study has limitations including the retrospective character, which generates a potential selection bias and the relatively low number of included patients. Being a single-center study, the results are noted to be vulnerable to the specific surgical and medical skills at that particular clinic. The lack of differentiation between entities of MSGC is acknowledged as a limitation, potentially leading to false implications for some specific entities. Despite these limitations, the explorative nature of the study is emphasized, as it is the first to analyze the impact of tumor budding on MSGC in terms of 5-YSR, 5-YDFSR mean OS, mean DFS, tumor size, nodal status, and recurrence. The authors stress the need for further studies in larger patient collectives with differentiation of tumor entities to fully understand the influence of tumor budding and its potential as a prognostic factor in the treatment of MSGC.

Current trends in oncological treatment of MSGC favor a less invasive surgical approach in terms of primary resection of mostly parotideal carcinomas. Elective neck dissection is not carried out in cN0 staged patients unless risk factors are detected during staging or histopathological examination. These risk factors include locally advanced tumors (T3/4), high-grade entities and lymphangioinvasion. Recognizing these risk factors leads to further personalized oncological treatment and is crucial to prevent undertreatment and recurrences. The findings support the hypothesis of tumor budding as a prognostic factor in MSGC, urging further exploration for enhanced therapy of patients to prevent recurrences and improve overall survival.

## Data availability statement

The original contributions presented in the study are included in the article/supplementary material. Further inquiries can be directed to the corresponding author.

## Ethics statement

The studies involving humans were approved by Ethik-Kommission der Albert-Ludwigs-Universität Freiburg. The studies were conducted in accordance with the local legislation and institutional requirements. The participants provided their written informed consent to participate in this study.

## Author contributions

VB: Conceptualization, Data curation, Formal analysis, Investigation, Writing – original draft, Writing – review & editing. GK: Conceptualization, Investigation, Methodology, Writing – original draft, Writing – review & editing. TV: Conceptualization, Data curation, Investigation, Writing – original draft, Writing – review & editing. CB: Conceptualization, Investigation, Supervision, Writing – original draft, Writing – review & editing.
